# A Network-Based Approach to Explore the Mechanism and Bioactive Compounds of Erzhi Pill against Metabolic Dysfunction-Associated Fatty Liver Disease

**DOI:** 10.1155/2020/7867245

**Published:** 2020-07-16

**Authors:** Shaojie Huang, Fei Mu, Fei Li, Wenjun Wang, Haixia Chen, Lu Lei, Yang Ma, Yi Ding, Jingwen Wang

**Affiliations:** Department of Pharmacy, Xijing Hospital, Fourth Military Medical University, Xi'an, 710032, China

## Abstract

Erzhi pill (EZP), a classical traditional Chinese medicine prescription, exerts a potent hepatoprotective effect against metabolic dysfunction-associated fatty liver disease (MAFLD), previously known as nonalcoholic fatty liver disease (NAFLD). However, the mechanism and bioactive compounds underlying the hepatoprotective effect of EZP have not been fully elucidated. In this study, a systematic analytical platform was built to explore the mechanism and bioactive compounds of EZP against MAFLD. This was carried out through target prediction, protein-protein interaction (PPI) network construction, gene ontology, KEGG pathway enrichment, and molecular docking. According to the topological parameters of the PPI network, compound-target-pathway network, 9 targets, and 11 bioactive compounds were identified as core targets and bioactive compounds for molecular docking. The results showed that EZP exerts anti-MAFLD effects through a multicomponent, multitarget, multipathway manner, and luteolin and linarin may be the bioactive compounds of EZP. This study provides further research insights and helps explore the hepatoprotective mechanism of EZP.

## 1. Introduction

Metabolic dysfunction-associated fatty liver disease (MAFLD), previously known as nonalcoholic fatty liver disease (NAFLD) [1, 2], is defined as the presence of liver cells with steatosis exceeding 5% and the lack of secondary causes of liver fat accumulation, such as drinking of alcohol [3]. With dramatic lifestyle modifications, the MAFLD has developed into a global health concern over the past decades [4]. Moreover, studies have increasingly shown the multisystem disease nature of MAFLD which affects several organs and increases the risk of type 2 diabetes and cardiovascular, cardiac, and chronic kidney diseases [5, 6]. Significant weight loss and change of dietary habits will have a salutary effect on MAFLD; however, new treatment strategies are urgently needed [7]. The reason is that with the changing dietary habits and lifestyle, MAFLD is one of the most important causes of liver disease worldwide. More importantly, MAFLD may eventually become the primary cause of end-stage liver disease [4]. Therefore, there is an urgent need for safe and effective drugs against MAFLD.

Previous studies have shown that some traditional Chinese medicine (TCM) formulaes, such as Dachaihu decoction, have good efficacy against MAFLD [8]. Erzhi pill (EZP) is a TCM used for liver disease in the past centuries. EZP consists of Ligustri Lucidi Fructus (LLF) and Ecliptae Herba (EH) at a ratio of 1 : 1 and functions as a liver and kidney tonic in traditional Chinese medicine theory. A previous study showed the hepatoprotective effect of EZP by the antioxidative defense system enhancement and the inflammatory response through the TSC/mTOR signaling pathway [9]. EZP has also been used to treat diabetes and metabolic syndrome. However, studies on the mechanism of EZP against MAFLD are still lacking.

The network pharmacology presented in 2008 [10] has holistic and systematic research methods and characteristics of focusing on the interaction between drugs and the body system. This is consistent with the characteristics of multiple targets and multiple pathways in TCM [11], becoming an efficient tool to systematically analyse the multiple targets and multiple pathway mechanisms of TCM. Several studies that employed network pharmacology to investigate the mechanism of TCM have been successful [12, 13]. In addition, the interaction of compounds, targets, and pathways can be established with network pharmacology, which helps identify potential bioactive compounds and pathways of TCM.

In this study, a systematic analytical platform for predicting potential bioactive compounds, targets, and molecular mechanisms of EZP against MAFLD was built. Detailed methods included potential bioactive compound collection, EZP- and MAFLD-related target prediction, protein-protein interaction (PPI) network construction, gene ontology and pathway enrichment, and molecular docking. This study provides a further research direction for the exploration of the hepatoprotective mechanism of EZP.

## 2. Materials and Methods

### 2.1. Extraction of Bioactive Components of EZP

The date for EZP compounds were mainly obtained from the Traditional Chinese Medicine Systems Pharmacology Database (TCMSP, http://lsp.nwu.edu.cn/tcmsp.php, Version 2.3), a pharmacology platform that provides information on drugs, targets, and diseases, by retrieving *Fructus Ligustri Lucidi* and *Ecliptae Herba*. Twelve absorption, distribution, metabolism, and excretion- (ADME-) related parameters of herbal ingredients were also extraction from the TCMSP [14]. Considering that oral administration of EZP, OB [15], and DL [16] was used for identifying bioactive compounds of EZP, the components with OB ≥ 30% and DL ≥ 0.18 were identified as potential bioactive compounds.

### 2.2. Collection of Potential Targets of the Bioactive Compounds of EZP

Bioactive compound-related targets were obtained from PharmMapper (http://lilab-ecust.cn/pharmmapper/, Version 2017), TargetNet (http://targetnet.scbdd.com/home/index/), and Swiss Target Prediction (http://www.swisstargetprediction.ch/, 2019 version) [17–22]. These platforms achieved compound-related targets by adopting various prediction algorithms. First, information on the bioactive compounds was collected from PubChem (https://pubchem.ncbi.nlm.nih.gov/) and TCMSP, including molecular structures in mol2 format and canonical smiles. To predict targets of compounds, molecular structures in mol2 were uploaded to PharmMapper with limitation to “Homo sapiens” and a normal fit score ≥ 0.6. In addition, canonical smiles were uploaded to Swiss Target Prediction and TargetNet with limitation to “Homo sapiens” (in Swiss Target Prediction), AUC ≥ 0.7 (in TargetNet), and probability ≥ 0.6. Finally, all the targets were transferred to UniProtKB (https://www.uniprot.org/) to avoid mix-up across the databases and platforms.

### 2.3. Construction of the MAFLD Target Database

Considering the different advantages and characteristics of each database, four databases were used to collect the MAFLD-related targets. By retrieving “nonalcoholic fatty liver disease” in GeneCards (https://www.genecards.org/), DrugBank (https://www.drugbank.ca/, version 5.1.5), Online Mendelian Inheritance in Man (OMIM, http://omim.org/, updated on Jan. 15, 2019), and National Centre for Biotechnology Information Gene (NCBI Gene, https://www.ncbi.nlm.nih.gov/gene/) MAFLD-related targets were retrieved. All the four databases are freely accessible platforms that contain comprehensive molecular information about drugs, targets, targets related to disease, gene function, etc. and can be used to collect targets related to the disease [23–26]. To maintain the reliability of the target collection, only the targets approved by the FDA in DrugBank, norm fit scores higher than 20 in GeneCards or the species limited to “Homo sapiens” in the NCBI Gene were identified as MAFLD-related targets. Finally, the target names were standardized to the UniProtKB form and duplicates were removed.

### 2.4. Construction of Protein-Protein Interaction (PPI) Network

A PPI network was built and analyzed by Search Tool for the Retrieval of Interacting Genes (STRING, https://string-db.org/), which can be employed for the system-wide understanding of cellular function between the expressed proteins [27]. After removing the overlap section and standardizing target names, the intersection of bioactive compound-related targets and MAFLD-related targets were uploaded to STRING with limitations to “Homo sapiens” and a confidence score > 0.9. The PPI network was constructed and visualized using Cytoscape 3.7.1, a software that is used for analyzing and visualizing biomolecular interaction networks [28].

### 2.5. Enrichment Analysis and Network Construction

Database for Annotation, Visualization, and Integrated Discovery (DAVID, https://david.nicifcrf.gov/, version 6.8) was used for enrichment analysis with the screening criteria of *P* ≤ 0.05 using Bonferroni correction [29]. Furthermore, KEGG Mapper (https://www.genome.jp/kegg/mapper.html) was employed for the analyses of upstream and downstream genes of the key signaling pathway [30, 31]. Thereafter, pathways with the top 20 protein numbers were used for the establishment of the compound-target-pathway network by Cytoscape.

### 2.6. Molecular Docking

Molecular docking was performed with AutoDock Tools [32] (version 1.5.6 http://mgltools.scripps.edu/). The 3D molecular structures of the bioactive compounds were collected from TCMSP in mol2 format and transformed into PDPQT format with AutoDock Tools. Protein Data Bank (PDB, http://www.rcsb.org/) was utilised for the collection of crystal structures of the core targets. AutoDock Tools were further used for removal of water and addition of hydrogen atoms to the crystal structures of core targets and saved as PDPQT format. Molecular docking between the bioactive compounds and core targets was performed with AutoDock. Finally, the binding pattern with the lowest binding energy was selected for further analysis. The interactions between the bioactive compounds and the core targets were visualized as 3D diagrams using PyMol 1.8.

## 3. Result

### 3.1. Bioactive Compounds in EZP

There were 166 compounds of EZP retrieved from TCMSP, including 47 in EH, and 119 in LLF, and 5 overlapping compounds were removed, resulting in 161 identified compounds. Finally, 20 bioactive compounds were identified after ADME screening with OB ≥ 30% and DL ≥ 0.18, 13 in LLF and 9 in EH (2 were duplicated and therefore removed). This is illustrated in Table 1. Some compounds that were removed after ADME screening have been identified as the main compounds of EZP in previous studies [33, 34]. Therefore, oleanolic acid, salidroside, and specnuezhenide were identified as bioactive compounds.

### 3.2. Potential Target Prediction for Bioactive Compounds of EZP

**Figure 1:**
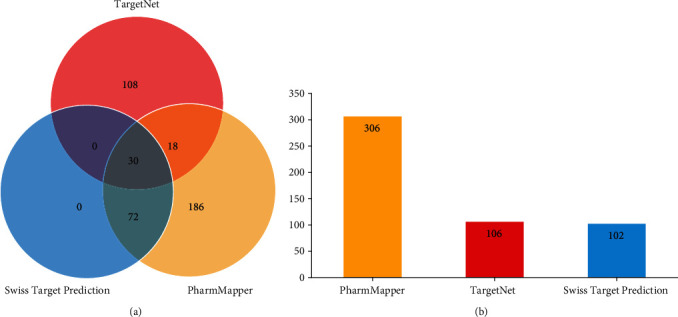
Analysis of predicted genes of potential bioactive compounds of EZP from 3 databases. (a) Venn graph showing the number of the overlapping genes from PharmMapper (yellow), TargetNet (red), and Swiss Target Prediction (blue). (b) The number of predicted genes from each database.

To identify potential targets of the 23 bioactive compounds, Swiss Target Prediction, PharmMapper, and TargetNet were used to predict the bioactive compounds' targets. There were 306 targets from PharmMapper (norm fit > 0.6), 156 targets from TargetNet (probability > 0.8), and 102 targets from Swiss Target Prediction (probability > 0.8) as shown in Figure 1(a). Finally, 30 targets were shared by all three databases, 72 targets were shared with Swiss Target Prediction and PharmMapper, and 18 targets were shared with PharmMapper and TargetNet (Figure 1(b)). After removal of duplicates, 414 targets were identified as potential targets of EZP for subsequent analysis. Detailed information on EZP-related targets is shown in Table S1.

### 3.3. Identification of Targets Related to MAFLD

**Figure 2:**
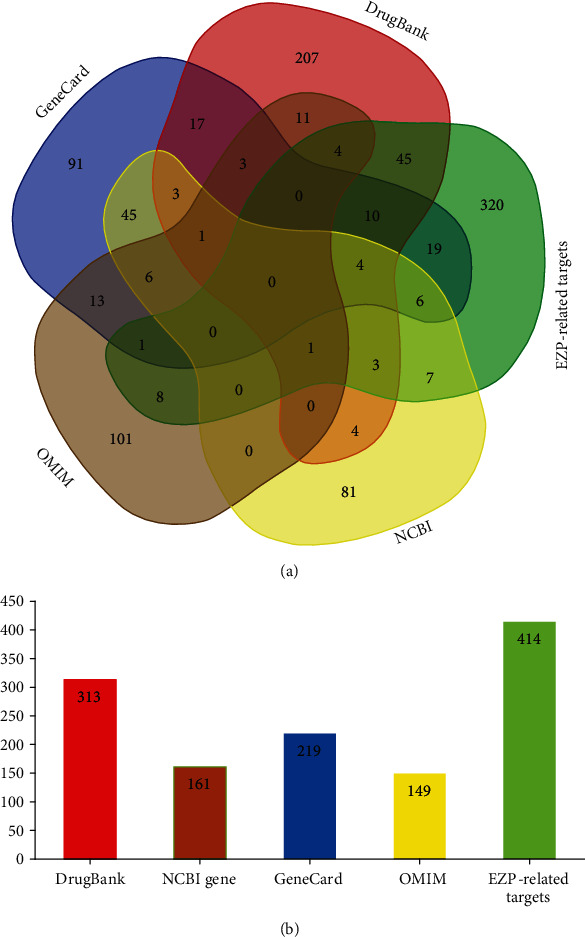
Analysis of predicted MAFLD genes. (a) Venn graph showing the numbers of overlapping genes from DrugBank (red), NCBI Gene (brown), GeneCard (blue), OMIM (yellow), and EZP-related targets (green). (b) The number of predicted genes from each database.

DrugBank, NCBI Gene, GeneCards, and OMIM were used to identify targets related to MAFLD. There were 313 targets from DrugBank, 161 targets from the NCBI Gene, 219 targets from GeneCards, and 149 targets from OMIM. After removal of duplicate targets, 691 targets were identified as potential therapeutic targets of MAFLD (Figure 2(b)). When overlapped with 414 targets of the EZP-related targets, 107 targets were found at the intersection of EZP-related targets and MAFLD-related targets (Figure 2(a)). Detailed information on MAFLD-related targets is presented in Table S2.

### 3.4. Protein-Protein Interaction Network

**Figure 3:**
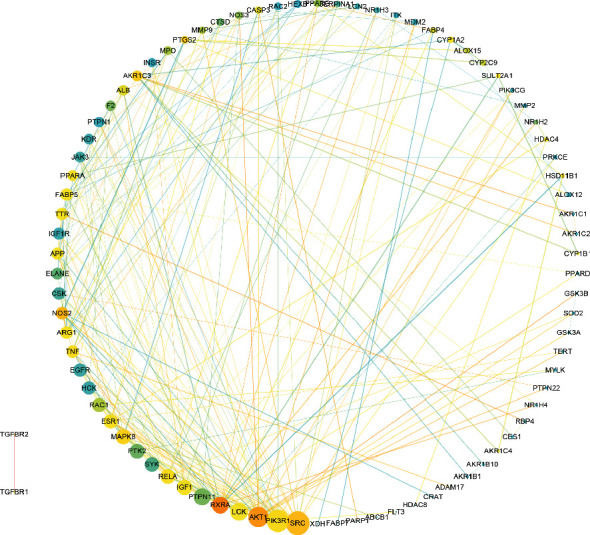
Visualization of the protein-protein interaction (PPI) using STRING and Cytoscape.

STRING and Cytoscape were used to analyze the interaction between the 107 common targets. The common targets were uploaded to STRING with limitation to “Homo sapiens” and a confidence score > 0.9. Then, the PPI network was established and visualized by Cytoscape 3.7.1 (Figure 3), which has 82 nodes and 247 edges. Network analyzer was used to calculate topological parameters of the PPI network for identifying the hub nodes and essential targets. In Figure 3, the size and color of the node were used to describe the topological parameters of the targets. The nodes with a larger degree were described by a larger size, and the nodes with bigger between centrality were described by a darker color. The overlap of the top 20 targets of degree, between centrality and closeness centrality, LCK, MAPK8, AKT1, RXRA, PIK3R1, SRC, RELA, ESR1, NOS2, and TNF were identified as hub nodes and essential targets of the PPI network.

### 3.5. GO and KEGG Pathway Enrichment Analyses

**Figure 4:**
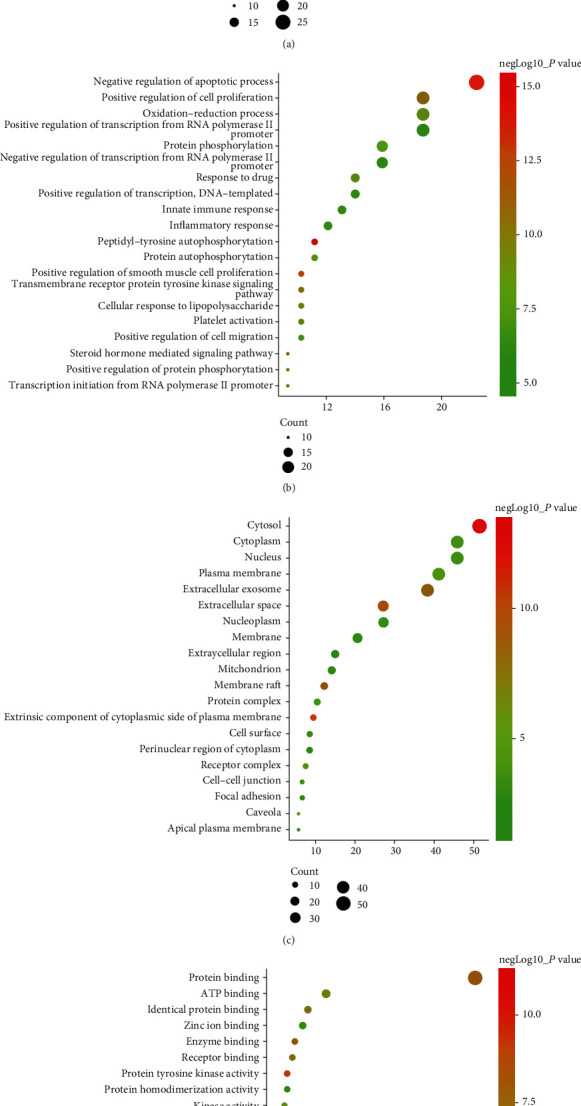
KEGG pathways and GO analyses. (a) KEGG pathway enrichment; (b) biological press (BP); (c) cellular component (c); (d) molecular function (MF).

DAVID was used for analyzing GO and KEGG pathway enrichment analyses to explore possible mechanisms of EZP against MAFLD by submitting 107 common targets to the database. The GO terms and KEGG pathways with *P* ≤ 0.05 were significantly enriched. Biological process (BP), cell compound (CC), and molecular function (MF) are the three components of GO term enrichment analysis. The top 20 enriched results were graphed using ImageGP (Figure 4). The results showed that 107 targets were significantly enriched in 72 BPs, 38 CCs, 103 MFs, and 83 pathways. Detailed information on GO and KEGG pathway enrichment analyses is presented in Table S3.

### 3.6. Construction of Compound-Target-Pathway Network

**Figure 5:**
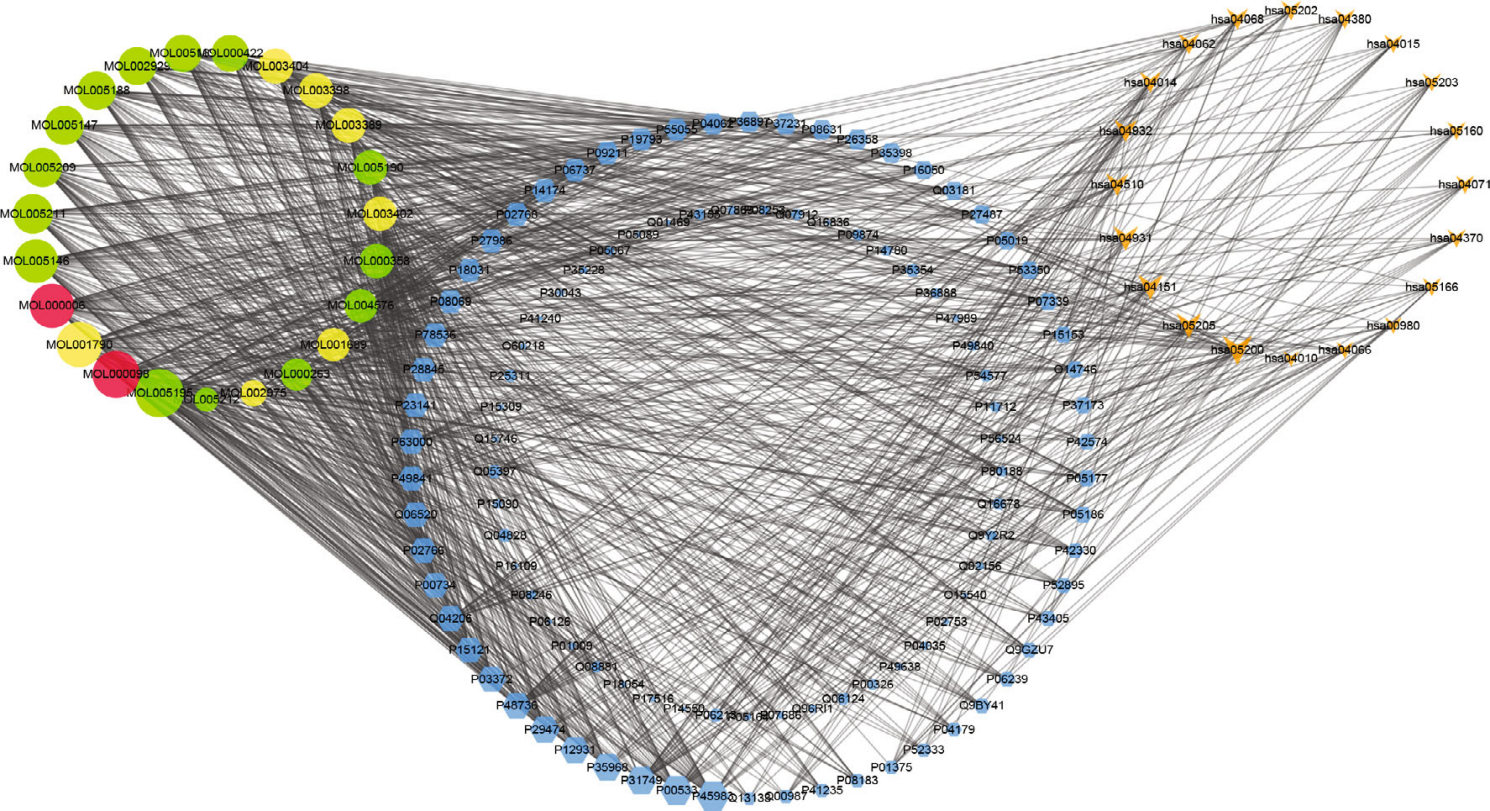
Compound-target-pathway network. Green circles represent bioactive components from LLF, yellow circles represent bioactive components from EH, red circles represent duplicated components of EH and LLF, blue hexagons represent putative targets, and orange V shapes represent the top 20 pathways.

According to the GO and KEGG pathway enrichment results, a compound-target-pathway network was established by Cytoscape (Figure 5). The compound-target-pathway network included 150 nodes and 1141 edges, circles represent bioactive components from EZP, green circles represent bioactive components from LLF, yellow circles represent bioactive components from EH, red circles represent duplicated components of EH and LLF, blue hexagons represent putative targets, and orange V shapes represent the top 20 pathway. In the compound-target-pathway network, 11 compounds had a higher than average degree, which showed that they played a pivotal role in the network. The 11 core compounds were MOL005195, MOL000098, MOL001790, MOL000006, MOL005146, MOL005211, MOL005209, MOL005147, MOL005188, and MOL002929. Targets are bridges between compounds and pathways. The interaction of the top 20 targets of the PPI network and the compound-target-pathway network was identified as core targets, which means that they play an essential role in both PPI network and compound-target-pathway network. Finally, nine targets, MAPK8, EGFR, AKT1, SRC, ESR1, RELA, RAC1, IGF1R, and PIK3R1 were identified as core targets.

### 3.7. Molecular Docking

**Figure 6:**
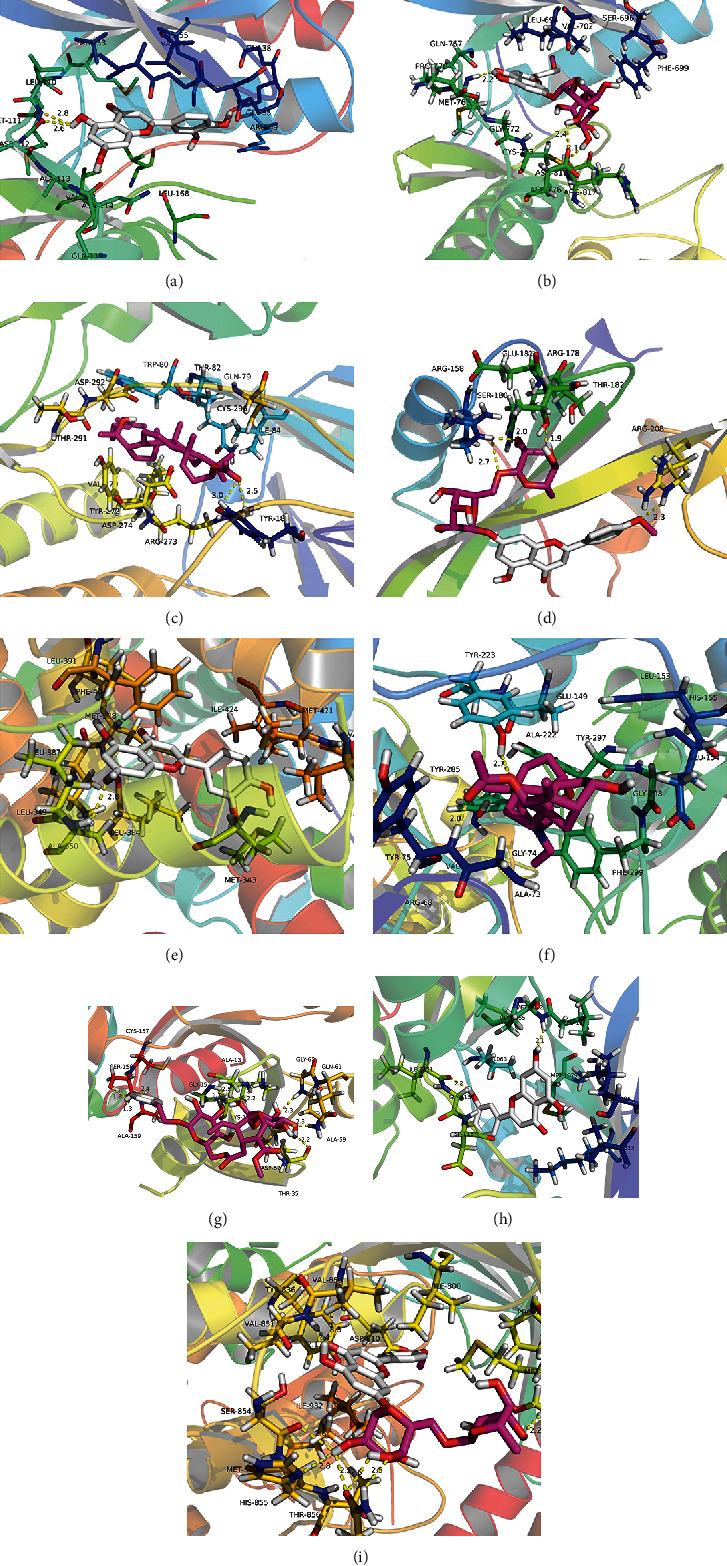
Molecular models of binding of bioactive compounds to the core targets. (a) MOL000006 to MAPK8; (b) MOL001790 to EGFR; (c) MOL005169 to AKT1; (d) MOL001790 to SRC; (e) MOL000006 to ESR1; (f) MOL005209 to RELA; (g) MOL005188 to RAC1; (h) MOL000006 to IGF1R; (i) MOL001790 to PIK3R1.

Docking studies were carried out between 11 core compounds and 9 core targets to test the reliability of the drug-target interaction. These targets were chosen as core targets because they play an essential role in the top 20 KEGG pathway, but they were also core targets of the PPI network, which means that these targets may be the center of the regulatory network of EZP against MAFLD. The binding energy and grid box are shown in Table 2. The results showed that there was a stronger interaction between MOL000006, MOL000098, MOL001790, MOL005160, MOL005188, and MOL005209 and core targets. The binding energy of some docking pattern was even lower than that of the original ligand, such as MOL000006 binding with MAPK8 and MOL001790 binding with EGFR and MOL005209 binding with RELA.  Figure 6 shows the docking patterns of bioactive compounds interacting with core targets in the lowest binding energy illustrated by PyMol, and the hydrogen bond is showed by a yellow imaginary line. The results showed that MOL000006 and MOL001790 have the lowest binding energy with 3 of the 9 core targets; MOL005169, MOL005188, and MOL005209 have the lowest binding energy with 1 of the 9 core targets, which means that these five compounds may have more important functions in the regulatory network of EZP against MAFLD.

## 4. Discussion

**Figure 7:**
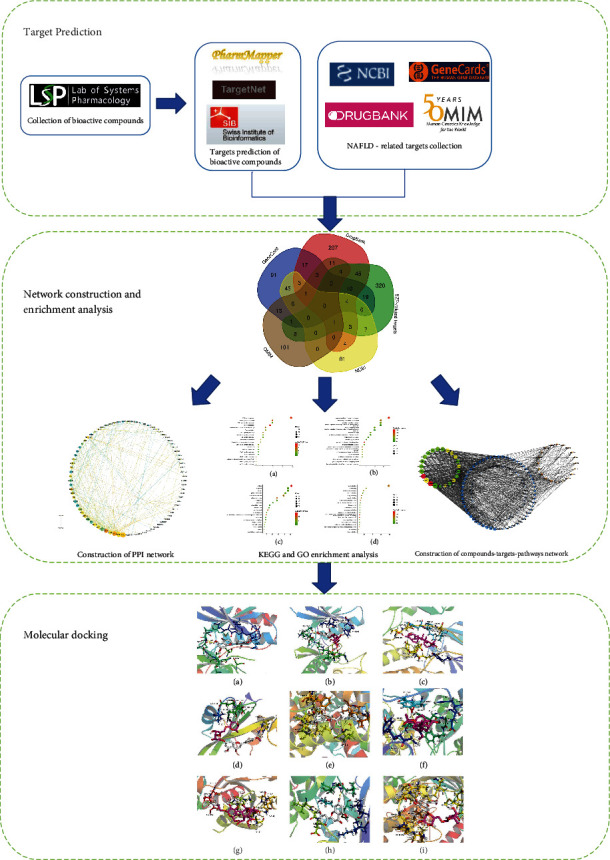
The experimental flow of this study.

In this study, the mechanism and bioactive compounds were investigated using a bioinformatics method to investigate the hepatoprotective effects of EZP. The results showed that 83 pathways and 72 biological processes were involved. According to the topological parameters of the compound-target-pathway network and the PPI network, 11 bioactive compounds and 9 core targets were identified. Finally, molecular docking was used to test the reliability of the drug-target interaction. The experimental flow is shown in Figure 7. This study could provide a better understanding of the hepatoprotective effect of EZP against MAFLD in a multicomponent and multitarget manner, which provides further insights for exploring the hepatoprotective mechanism of EZP.

In clinical treatment, EZP is administered orally. Hence, ADME-related paraments OB and DL were used for screening potential bioactive compounds of EZP. Then, the degree of potentially bioactive compounds of the compound-target-pathway network higher than average was used for a second screening. Eleven bioactive compounds were identified from EZP. To ensure the reliability of the target prediction, three different target identification databases and three multiple information sources were used to predict related targets. The PPI network and compound-target-pathway network were used to identify core targets of the regulatory network of EZP against MAFLD. The interaction of the top 20 targets of the PPI network and compound-target-pathway network was identified as a core target. Nodes with a high degree often play an essential role in the network. Core targets' degrees were higher in the PPI network and the compound-target-pathway network. This means that core targets were essential in the regulatory network of EZP against MAFLD.

The pathological mechanisms of MAFLD are complicated [35]. At present, it is a widely accepted theory that the capacity of the liver to handle the primary metabolic energy is overwhelming leading to accumulation of toxic lipid species that induce hepatocellular stress, injury, and death [35–37]. When the liver cannot handle excessive fatty acids, the excess may serve as substrates, leading to generation of lipotoxic species which would provoke ER stress and hepatocellular injury [38]. Hence, regulating fatty acid metabolism and declining hepatocellular stress, injury, and death induced by toxic lipid species are two aspects of MAFLD therapeutic strategies.

Nine core targets, MAPK8, EGFR, AKT1, SRC, ESR1 RELA, RAC1, IGF1R, and PIK3R1, were identified for molecular docking with 11 bioactive compounds. The results showed that the bioactive compounds of EZP have good affinity for nine core targets. These core targets play essential roles in the pathophysiology of MAFLD. The hsa04151: PI3K-Akt signaling pathway, in which AKT1 plays a pivotal role, was a significant result of KEGG pathway enrichment. This pathway has been proved to be closely related to the hepatoprotective effect of EZP via inhibition of hepatocyte apoptosis [39]. MAPK8 also acts a pivotal part of the development of MAFLD. During inflammation postreceptor insulin signaling is significantly impaired by MAPK8, which leads to the production of toxic lipid species and hepatocyte injury [40].

**Figure 8:**
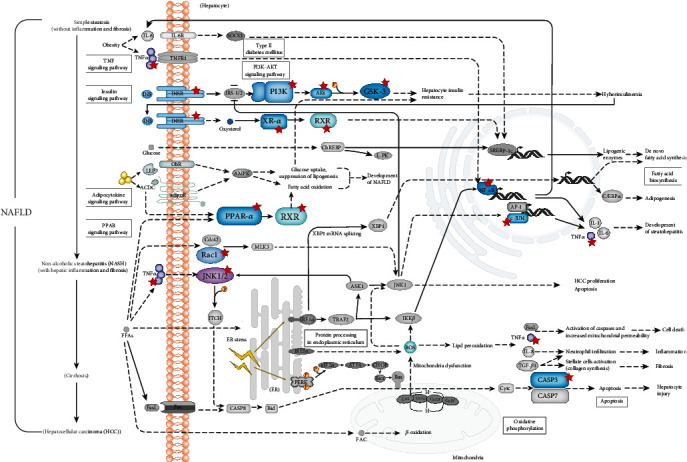
Stage-dependent progression of MAFLD. EZP-related targets are marked with red stars.

Metabolic syndrome (MetS) is the strongest risk factor for MAFLD. Among the MetS, diabetes is the clearest biological factor associated with MAFLD and 75% of patients with type 2 diabetes have MAFLD [41].  Figure 4 shows the 16 targets involved in hsa04931: Insulin resistance. Insulin resistance is a common feature of MAFLD and leads to improper release of fatty acids further impairing insulin signaling throughout the body [42]. Molecular docking also showed that the binding energy of bioactive compounds of EZP (except lucidusculine and olitoriside with IGF1R) was lower than -5 kcal/mol, suggesting that the bioactive compounds of EZP may exert anti-MAFLD effects by insulin resistance.  Figure 4 is a representation of 14 targets involved in hsa04932: Nonalcoholic fatty liver disease, which shows a stage-dependent progression of NAFLD. As shown in Figure 8, all 14 targets, marked with stars, play important roles in the progress of MAFLD, both in excess lipid accumulation and production of reactive oxygen species (ROS). This further leads to cytokine production, cell death promotion, inflammation and fibrosis. There were 14 targets enriched in hsa04932 including TNF, CASP3, MAPK, PPARA, RELA, and AKT1. These targets all play important roles in promoting cell death, inflammation, and fibrosis [43, 44], meaning that EZP may exert anti-MAFLD by these targets.

## 5. Conclusion

Overall, this study provides a theoretical basis for EZP exertion of an anti-MAFLD effect through a multicomponent, multitarget, and multipathway manner. In addition, we screened the bioactive compounds of EZP and tested them by molecular docking, providing a further understanding to explore the hepatoprotective mechanisms of EZP.

## Figures and Tables

**Table 1:** A list of bioactive compounds of EZP for network analysis.

Mol ID	Molecule name	MW	OB (%)	DL	Pubchem ID	Herb
MOL000006	Luteolin	286.25	36.16	0.25	5280445	LLF/EH
MOL000098	Quercetin	302.25	46.43	0.28	5280343	LLF/EH
MOL000263	Oleanolic acid	456.78	29.02	0.76	10494	LLF
MOL000358	Beta-sitosterol	414.79	36.91	0.75	222284	LLF
MOL000422	Kaempferol	286.25	41.88	0.24	5280863	LLF
MOL002929	Salidroside	300.34	7.01	0.20	159278	LLF
MOL004576	Taxifolin	304.27	57.84	0.27	439533	LLF
MOL005146	Lucidumoside D	568.63	48.87	0.71	10531060	LLF
MOL005147	Lucidumoside D_qt	406.47	54.41	0.47	—	LLF
MOL005169	(20S)-24-ene-3*β*,20-diol-3-acetate	486.86	40.23	0.82	185500	LLF
MOL005188	Specnuezhenide	686.73	19.30	0.50	91895359	LLF
MOL005190	Eriodictyol	288.27	71.79	0.24	440735	LLF
MOL005195	Syringaresinol diglucoside_qt	450.48	83.12	0.8	21603207	LLF
MOL005209	Lucidusculine	401.6	30.11	0.75	101286217	LLF
MOL005211	Olitoriside	696.87	65.45	0.23	94348	LLF
MOL005212	Olitoriside_qt	404.55	103.23	0.78	—	LLF
MOL001689	Acacetin	284.28	34.97	0.24	5280442	EH
MOL001790	Linarin	592.6	39.84	0.71	5317025	EH
MOL002975	Butin	272.27	69.94	0.21	28125525	EH
MOL003389	3′-O-Methylorobol	300.28	57.41	0.27	5489605	EH
MOL003398	Pratensein	299.27	39.06	0.28	5319744	EH
MOL003402	Demethylwedelolactone	302.25	72.13	0.43	5281803	EH
MOL003404	Wedelolactone	314.26	49.6	0.48	5281813	EH

**Table 2:** Results of molecular docking between bioactive compounds and core targets.

Target	Binding energy (kcal/mol)												Grid box		
	Ligand	MOL000006	MOL000098	MOL001790	MOL002929	MOL005146	MOL005147	MOL005169	MOL005188	MOL005195	MOL005209	MOL005211	*X*	*Y*	*Z*
MAPK8	-6.7	-8.0	-7.9	-7.7	-7.0	-6.2	-6.4	-6.5	-6.1	-6.5	-5.8	-4.9	21.09	9.691	31.723
EGFR	-8.3	-8.5	-8.5	-8.9	-7.1	-7.5	-7.5	-8.1	-7.7	-6.4	-8.3	-4.6	-51.608	-0.456	-22.4
AKT1	-12.9	-8.9	-8.9	-10.0	-8.0	-8.7	-8.4	-10.2	-8.6	-9.2	-5.7	-8.5	-11.688	-14.389	13.487
SRC	-6.4	-6.2	-5.8	-6.7	-5.6	-5.3	-5.3	-5.7	-6.3	-4.8	-5.7	-6.1	-1.417	20.293	-6.568
ESR1	-11.5	-8.7	-8.4	-3.0	-7.2	-6.8	-7.9	-2.7	-4.7	-3.3	-5.8	-0.4	15.683	22.798	33.284
RELA	-8.3	-8.8	-8.8	-8.8	-8.8	-8.9	-8.6	-7.6	-8.7	-7.5	-9.5	3.0	63.345	19.545	10.383
RAC1	-9.2	-7.1	-7.1	-7.3	-6.9	-6.7	-6.5	-6.7	-7.3	-5.6	-5.9	-6.1	17.263	-33.71	-23.989
IGF1R	-10.7	-7.7	-7.5	-3.4	-6.6	-6.5	-6.9	-5.7	-6	-7.0	0.7	5.9	7.48	1.242	21.691
PIK3R1	-10.7	-8.6	-8.6	-9.3	-7.1	-7.4	-7.0	-8.6	-7.7	-6.0	-7.9	-6.6	0.97	113.523	18.684

## Data Availability

The data used to support the findings of this study are available from the corresponding author upon request.

## Abbreviations

EZP: Erzhi pill
MAFLD: Metabolic dysfunction-associated fatty liver disease
NAFLD: Nonalcoholic fatty liver disease
PPI: Protein-protein interaction
NAFL: Nonalcoholic fatty liver
TCM: Traditional Chinese medicine
LLF: Ligustri Lucidi Fructus
EH: Ecliptae Herba
TCMSP: Traditional Chinese Medicine Systems Pharmacology Database
OMIM: Online Mendelian Inheritance in Man
NCBI Gene: National Centre for Biotechnology Information Gene
STRING: Search Tool for the Retrieval of Interacting Genes
DAVID: Database for Annotation Visualization and Integrated Discovery
GO: Gene ontology
KEGG: Kyoto Encyclopedia of Genes and Genomes
MAPK8: Mitogen-activated protein kinase 1
EGFR: Epidermal growth factor receptor
AKT1: RAC-alpha serine/threonine-protein kinase
SRC: Protooncogene tyrosine-protein kinase Src
ESR1: Estrogen receptor
RELA: Transcription factor p65
RAC1: Ras-related C3 botulinum toxin substrate 1
IGF1R: Insulin-like growth factor 1 receptor
PIK3R1: Phosphatidylinositol 3-kinase regulatory subunit alpha
TNF: Tumor necrosis factor
CASP3: Caspase-3
PPARA: Peroxisome proliferator-activated receptor alpha
LCK: Tyrosine-protein kinase Lck
RXRA: Retinoic acid receptor RXR-alpha
NOS2: Nitric oxide synthase
BP: Biological process
CC: Cell compound
MF: Molecular function
MetS: Metabolic syndrome
ROS: Reactive oxygen species.

## Supplementary Materials

Supplementary Materials Table S1: putative targets for bioactive compounds contained in EZP. There were 306 targets from PharmMapper, 156 targets from TargetNet, and 102 targets from Swiss Target Prediction. Table S2: targets related to NAFLD. Detailed information of targets related to NAFLD; there were 313 targets were from DrugBank, 161 targets from NCBI Gene, 219 targets from GeneCard, and 149 targets from OMIM. Table S3: the results of GO and KEGG pathway enrichment analyses. 107 targets were significantly enriched in 72 BPs, 38 CCs, 103 MFs, and 83 pathways.
