# Supplementary material for: A Network-Based Approach to Explore the Mechanism and Bioactive Compounds of Erzhi Pill against Metabolic Dysfunction-Associated Fatty Liver Disease
Source: J Diabetes Res. 2020 Jul 16;2020:7867245. doi: 10.1155/2020/7867245 (PMC7382733; doi:10.1155/2020/7867245)
Supplement: Supplementary Materials — Table S1: putative targets for bioactive compounds contained in EZP. There were 306 targets from PharmMapper, 156 targets from TargetNet, and 102 targets from Swiss Target Prediction. Table S2: targets related to NAFLD. Detailed information of targets related to NAFLD; there were 313 targets were from DrugBank, 161 targets from NCBI Gene, 219 targets from GeneCard, and 149 targets from OMIM. Table S3: the results of GO and KEGG pathway enrichment analyses. 107 targets were significantly enriched in 72 BPs, 38 CCs, 103 MFs, and 83 pathways. [file 7867245.f1.zip › Description of supplementary materials.pdf]

Description of each supplementary material file

Table S1 Putative targets for bioactive compounds contained in EZP. There were 306 targets from PharmMapper, 156 targets from TargetNet and 102 targets from Swiss Target Prediction.

Table S2 Targets related to MAFLD. Detailed information of targets related to MAFLD, there were 313 targets were from DrugBank, 161 targets from NCBI gene, 219 targets from GeneCards and 149 targets from OMIM.

Table S3 The results of GO and KEGG pathway enrichment. 107 targets were significantly enriched in 72 BPs, 38 CCs, 103 MFs and 83 pathways.
